# Effects of nitrate in combination with lactate on rumen fermentation and methane production in an *in vitro* batch culture

**DOI:** 10.5713/ab.25.0020

**Published:** 2025-04-11

**Authors:** Beomseok Bae, Logan Pope, Kiyeon Park, Chanhee Lee

**Affiliations:** 1 Department of Animal Sciences, The Ohio State University, Wooster, OH, USA

**Keywords:** Nitrate Reduction, Nitrate Toxicity, Volatile Fatty Acids

## Abstract

**Objective:**

This *in vitro* study determined the combination effect of nitrate and lactate on nitrate reduction, rumen fermentation and methane production. We hypothesized that the combination decreases methane production and enhances nitrate reduction.

**Methods:**

An *in vitro* batch culture was conducted to determine the 2 main factors of nitrate and lactate in 2×2 factorial arrangement: a basal diet (CON), the basal diet supplemented with nitrate (2.2% in dietary dry matter [DM]; Nit), lactate (2.2%; Lact), or the combination of nitrate and lactate (Nit-Lact). Rumen fluids from lactating Holstein cows were obtained and mixed with McDougall’s buffer for the *in vitro* batch culture. Total gas production, methane production, pH, volatile fatty acid (VFA), NH_3_-N, NO_3_-N and NO_2_-N concentration, and DM digestibility were determined.

**Results:**

A trend of interaction between nitrate and lactate was observed for DM digestibility (p = 0.06), which occurred because DM digestibility tended to decrease for Nit compared with CON (42.2 vs. 45.3%) but did not for Nit-Lact. Nitrate decreased (p<0.05) methane (25.6 vs. 31.0 mL at 24 h) and total gas production (296 vs. 313 mL at 24 h). Total VFA production was not affected by treatments. However, nitrate increased the proportion of acetate (p<0.01) and decreased (p<0.01) proportions of propionate, butyrate and valerate at 24 h of incubation. However, lactate increased valerate at 24 h of incubation (p<0.01). The concentration of NO_3_-N for Nit-Lact was lower than that of Nit (41.2 vs. 51.0 mg/L; p<0.01) at 3 h of incubation.

**Conclusion:**

Nitrate decreased methane production. The combination of nitrate and lactate had beneficial effects on DM digestibility and nitrate reduction, compared with nitrate alone although no interaction between nitrate and lactate was shown for VFA production and methane production. Changes in the major VFA by nitrate indicated that nitrate acted as an alternative electron acceptor to decrease methane production.

## INTRODUCTION

Methane is one of the main greenhouse gases (GHG) that has strong global warming potential, i.e., 28 times greater than CO_2_ [1]. In the US, methane represented 11.5% of total GHG emissions in 2021, and 26.4% of total anthropogenic methane resulted from enteric fermentation of ruminants. Enteric fermentation was the largest anthropogenic source of the methane emitted in the US and the contribution has gradually increased, i.e., 6.5% from 1990 to 2021 [2].

Methane is produced as a natural end product of anaerobic fermentation in the rumen, where methanogenic archaea use electrons from H_2_ to reduce CO_2_ [3]. In addition to potential contribution to climate change, methane also represents a loss of energy in ruminants, which accounts for about 6% of gross energy [4]. Consequently, extensive research has been conducted to develop strategies to mitigate enteric methane emissions from ruminants [5]. In the nutritional aspect, various compounds have been explored to reduce enteric methane emissions [6]. However, the effectiveness of the compounds was often variable between studies. Moreover, some additives that showed methane mitigation failed to have long-term effectiveness due to microbial adaptation [6,7]. Therefore, the necessity to develop effective strategies in the long-term has been emphasized. In this context, nitrate (NO_3_) is one of the few promising feed additives that are strongly effective and persistent in methane mitigation [7].

Nitrate introduced into the rumen is reduced to nitrite (NO_2_) and then reduced to ammonium ion by microbes. Thermodynamically, the process of the nitrate reduction is more favorable than the reduction of CO_2_ to methane [8]. Therefore, nitrate acts as an alternative hydrogen sink competing with methanogens, resulting in lowering methane formation. Several studies have shown anti-methanogenesis effects of nitrate in ruminants [7,9,10]. However, feeding nitrate as a feed additive has been a concern because of potential nitrate toxicity. If nitrate is not completely reduced to ammonia, leading to nitrite accumulation in the rumen, nitrite is absorbed into blood and forms methemoglobin which limits O_2_ supply to cells in the body [11]. Depending on the degree of methemoglobin formed in blood, it can cause depressed feed intake and production, increased respiration and urination, weakness, ataxia, reproductive failure, hypersensitivity, cyanosis of the tongue, blood discoloration, and even death [11,12]. Because of the potential health concerns, nitrate has not been approved as a feed additive for ruminant animals in the US, despite its strong ability of methane mitigation. Furthermore, nitrate and nitrite toxicity can induce depression of rumen microbial growth and decrease carbohydrase activities [13]. Some studies also indicated indirect negative effects of feeding nitrate to ruminants i.e., lowering forage digestibility, toxic effects on cellulolytic microbes, and altering the ratio of volatile fatty acid (VFA) [12].

Nitrate in the rumen is reduced by nitrate reductase from various rumen microbes [14]. It was reported that nitrate and nitrite reducing bacteria, e.g., *Selenomonas ruminantium* have the ability to utilize lactate where electrons from lactate utilization are used to reduce nitrate and nitrite [15]. This indicates that the presence of lactate in the rumen might be helpful for complete reduction of nitrate to ammonia by rumen microbes, consequently lowering the risk of nitrate toxicity.

We hypothesized that nitrate supplementation will decrease methane production but lower dry matter (DM) digestibility and total gas production in an *in vitro* batch culture. However, supplying the combination of nitrate and lactate would alleviate the negative effects of nitrate due to rapid nitrate and nitrite reduction. The objective was to determine nitrate and nitrite concentrations, methane production, and rumen fermentation in an *in vitro* batch culture when nitrate was provided with or without lactate.

## MATERIALS AND METHODS

All procedures involving animals in this study were approved by The Ohio State University Institution of Animal Care and Use Committee (IACUC: 2017A00000069-R2).

### Experimental design

The experiment was conducted with 4 dietary treatments in a 2×2 factorial arrangement: 1) 1 g of a basal diet with 9.7 mg of urea (CON), 2) 1 g of the basal diet with 27 mg of sodium nitrate (2.2% of nitrate in dietary DM; Nit), 3) 1 g of the basal diet with 25 mg of sodium lactate and 9.7 mg of urea (2.2% of lactate in dietary DM; Lact), and 4) 1 g of the basal diet with 27 mg of sodium nitrate and 25 mg of sodium lactate (Nit-Lact). Sodium nitrate and sodium lactate were purchased from Sigma Aldrich (St. Louis, MO, USA). Urea was included for CON and Lact so that all treatment diets were isonitrogenous.

### *In vitro* batch culture

Two cannulated lactating Holstein cows at Krauss Dairy Research Center (Wooster, OH, USA) were used as donors of rumen fluids. Cows were housed in tie-stalls and fed a lactation diet (40% corn silage, 15% alfalfa haylage, and 45% concentrate on a DM basis) as a total mixed ration. The diet was offered *ad libitum* every morning and cows had free access to water. Rumen contents were collected from cranial, ventral, and dorsal sacs of the rumen 2 h after feeding. The rumen contents collected from the 2 cows were combined and strained through 2 layers of cheesecloths to obtain rumen fluid into a pre-warmed insulated flask. The rumen fluid was immediately transported to the laboratory and was further strained through 8 layers of cheesecloths.

McDougall’s buffer [16] containing resazurin was prepared and bubbled with CO_2_ gas in a water bath at 39°C to remove O_2_. The rumen fluid prepared was mixed with the buffer at a ratio of 1:3, and 60 mL of the buffered rumen fluid was distributed into 125 mL serum bottles containing the treatment diets, under a continuous stream of CO_2_. The bottles were sealed with rubber stoppers and aluminum seal caps. Then, they were incubated in a shaking incubator (100 rpm) at 39°C (Lab-Line Orbit Environ-Shaker 3527; Lab-Line Instruments Inc., Melrose Park, IL, USA). Therefore, a total of 56 serum bottles was used for 0, 0.5, 1, 3, 6, 12 and 24 h of incubation where samples were collected for gas and methane production (3, 6, 12, and 24 h), rumen fermentation (pH, NH_3_-N, and VFA; 3, 6, and 24 h), and nitrate and nitrite concentration (0, 0.5, 1, 3, 6, 12 and 24 h). Another set of 8 serum bottles were added and used only to measure DM digestibility (24 h). Therefore, the incubation included a total of 64 serum bottles and was repeated, i.e., a total of 2 incubations.

### Sampling and measurements

The basal diet used in the current study was the control diet used in a study by Porter et al [17], and the ingredients and chemical composition of the diet are shown in Table 1. The detailed procedures of chemical analyses can be found in Porter et al [17]. The basal diet was dried at 55°C for 3 d and ground through a 1-mm sieve. The dried and ground diet was weighed out into serum bottles and used as a substrate for the batch culture incubation.

Total gas production was determined by using a gas pressure manometer (VWR Manometer Pressure/Vacuum Gauges; VWR, Inc., Radnor, PA, USA) at each time point. Gas samples were collected from serum bottles into the pre-evacuated tube, and later a subsample of the gas was manually injected into gas chromatography (Hewlett-Packard 5890 series; Agilent Technologies, Santa Clara, CA, USA) using a gas-tight syringe (Hamil-ton 1700 series syringe 1710; Hamilton Company, Reno, NV, USA) to determine methane concentration. After gas sampling, serum bottles were opened, and pH was measured with a pH meter (Accumet Research AR25; Fisher Scientific, Pittsburgh, PA, USA), and then serum bottles were placed on ice immediately. The fermentation medium in each serum bottle were transferred to centrifuge tubes and centrifuged at 20,000×g for 14 min at 4°C. Supernatants were sampled for VFA, NH_3_, or nitrate and nitrite analysis. An aliquot was assayed for NH_3_ concentration [18] and VFA composition using gas chromatography (Hewlett-Packard 5890 series; Agilent Technologies). Another aliquot was assayed for nitrate and nitrite concentrations using a nitrate/nitrite colorimetric assay kit (Cayman Chemical Company, Ann Arbor, MI, USA).

A separate set of 8 serum bottles was used only for DM digestibility at 24 h of incubation. The entire medium in each serum bottle was filtered through a filter paper (Whatman 541, GE Healthcare Life Sciences, Chicago, IL, USA). The filtrate from each bottle was dried at 55°C for 48 h. Then, the DM digestibility was calculated from the weight of filtrate and the original weight of the substrate.

### Statistical analysis

All data were analyzed using the MIXED procedure of SAS (version 9.4; SAS institute Inc., Cary, NC, USA) as a randomized complete block design. Nitrate and lactate treatments and their interaction were used as fixed effects. Incubation was used as a random effect. The following statistical model was used:


$${\text{Y}}_{\text{ijk}}=\mu +{\text{Nitrate}}_{\text{i}}+{\text{Lactate}}_{\text{j}}+{\left(\text{Nitrate}\times \text{Lactate}\right)}_{\text{ij}}+{\text{I}}_{\text{k}}+{\text{e}}_{\text{ijk}}$$

where Y_ijk_ = dependent variables, μ = overall mean, Nitrate_i_ = fixed effect of nitrate supplementation, Lactate_j_ = fixed effect of lactate supplementation, (Nitrate × Lactate)_ij_ = fixed effect of interaction between nitrate and lactate, I_k_ = random effect of incubation, and e_ijk_ = residual error. Therefore, the model determined the main effects of nitrate or lactate supplementation and their interaction. For nitrate and nitrite concentrations at each time point during the 24-incubation, posterior comparisons were conducted because changes in nitrate and nitrite concentrations during the incubation between Nit and Nit-Lact was the interest. When an interaction between nitrate and lactate or posterior analysis were significant, the PDIFF option generated pairwise comparisons among treatments (i.e., Fisher’s protected least significant difference). Mean differences were considered significant when p<0.05 and tendency when 0.05≤p<0.10.

## RESULTS AND DISCUSSION

Nitrate supplementation decreased both methane and total gas production (p<0.01; Table 2) at all time points during the incubation except for total gas production at 3 h of incubation. Methane mitigation by nitrate supplementation was investigated by numerous studies [6,7,9,10]. As nitrate is reduced to ammonia, it accepts electrons from hydrogen. This process is thermodynamically more favorable than methanogenesis, making nitrate as an alternative hydrogen sink [7,8]. In the current study, nitrate decreased methane production by 18% at 24 h of incubation, regardless of the addition of lactate. This result aligns with previous *in vitro* studies which reported methane mitigation with nitrate supplementation [19,20]. In a meta-analysis conducted by Lee and Beauchemin [7], nitrate supplementation effectively and persistently decreased methane emissions from ruminants in a dose-dependent manner. Another meta-analysis reported that an increase of 1 g/kg of DM nitrate decreased methane production by 0.9% in dairy and beef cattle [9]. In the current study, however, although nitrate decreased methane production, the decreased methane occurred partly due to the decrease in total gas production. At 24 h of incubation, nitrate supplementation decreased total gas production and methane by 6% and 18%, respectively, suggesting that nitrate as an alternative electron acceptor decreased methane by about 13%. Braidot et al [21] reported that a high dose of nitrate supplementation suppressed total gas and methane-free gas production as well as methane production. Additionally, decreased DMI and animal productivity have been reported in previous *in vivo* studies when nitrate was fed to ruminant animals [22,23]. No synergistic effect was observed between nitrate and lactate supplementation on methane mitigation during the 24-h incubation. This suggests that the quantity of nitrate reduction to nitrite and ammonia did not differ significantly during the incubation when the combination of nitrate and lactate was provided compared with nitrate alone. For total gas production, lactate supplementation tended to suppress (p = 0.09) gas production at 6 h of incubation and suppressed it at 12 h of incubation (p<0.01). However, the decreased gas production for lactate supplementation did not occur at 24 h of incubation, suggesting the impact of lactate on overall feed fermentation was trivial. This is also, at least in part, supported by no effect of lactate supplementation on DM digestibility at 24 h of incubation.

It is interesting that a tendency of interaction between nitrate and lactate supplementation was observed for DM digestibility (p = 0.06; Table 2) at 24 h of incubation. The interaction tended to occur because DM digestibility was greater (p<0.05) for the Nit-Lact treatment compared with Nit. According to the interaction tendency and a numerical decrease in DM digestibility for Nit compared with CON (42.2% vs. 45.3%), it is likely that nitrate supplementation alone depressed feed digestion, which is supported by the decreases in total gas production. However, a decrease in DM digestibility did not occur when nitrate was provided with lactate, i.e., Nit-Lact versus CON. The trend of interaction for DM digestibility is difficult to explain because it did not occur for total gas production. We observed numerical and significant decreases in nitrate concentration for Nit-Lact compared with Nit at 1 and 3 h of incubation, respectively and greater nitrite concentration (p<0.05) for Nit-Lact compared with Nit at 1 and 3 h of incubation (Table 3). This suggests that the rate of nitrate reduction was likely greater when nitrate was provided with lactate compared with nitrate alone. The greater rate of nitrate reduction may have led to the lack of suppression on DM digestibility for Nit-Lact compared with Nit. However, still it does not explain no interaction between nitrate and lactate on total gas production and requires further studies.

No difference in rumen pH was observed in all treatments (Table 4). The supplementation of lactate tended to increase pH at 12 h of incubation, which did not occur at 3 and 24 h of incubation. Ammonia concentration for nitrate supplementation was lower (p<0.01) at 3 and 24 h or tended to be lower (p = 0.08) at 12 h of incubation compared with no nitrate supplementation, which is obvious because urea was added to CON and Lact for the isonitrogenous condition among treatments. The difference in ammonia concentration between nitrate and no nitrate supplementation was maintained during the 24-h incubation, which consistent with the finding by Lee et al [20]. This may suggest that nitrate provided was reduced through the assimilatory reduction process [12], and microbial utilization of nitrate as nonprotein nitrogen (NPN) may have been more efficient than that of urea or ammonia. However, lactate effect or interaction between nitrate and lactate were not observed for ammonia concentration during 24 h of incubation, suggesting that nitrate was utilized as NPN by most microbial organisms rather than just nitrate-reducing bacteria, such as *Veillonella parvula* [24,25].

No interaction for total VFA concentration or proportions of individual VFA indicates that there was no synergistic or antagonistic relationship between nitrate and lactate supplementation on VFA production (Table 4). While total VFA concentration did not differ among treatments, the composition of individual VFA was affected by treatments. Nitrate supplementation increased the proportion of acetate (p≤0.02) and decreased that of propionate and butyrate (p≤0.02) during the 24-h incubation. The increase in acetate and decrease in propionate for nitrate supplementation agree with previous study [20]. Thermodynamically, nitrate reduction is more favorable than propioneogenesis and methanogenesis. Therefore, nitrate likely decreased the production of propionate by acting as an alternative hydrogen sink and shifted rumen fermentation toward acetogenesis, an H_2_-releasing process [26,27]. Most cellulolytic bacteria encode genes for acetate production to generate adenosine triphosphate and release either H_2_ or formate to recycle electron carriers. In contrast, the production of longer-chain VFA like butyrate and valerate requires H_2_ when they are synthesized from acetyl-CoA or propionate [14]. Because acetogenesis may have been favored to support electron recycling for nitrate supplementation in the current study, metabolic pathways that rely on H_2_ utilization, such as the production of propionate, butyrate, and valerate, were likely suppressed. Lactate supplementation did not affect proportions of the major VFA except that the proportion of valerate increased (p≤0.05) during the 24-h incubation. Lactate in the rumen is primarily utilized to produce acetate, propionate and valerate [14,28]. and was likely utilized for valerate production in the current study. Proportion of iso-butyrate, 2-methyl butyrate, and iso-valerate were not affected by nitrate or lactate supplementation, suggesting that their effects on protein degradation were minimal.

It was obvious that nitrate supplementation increased NO_3_-N concentration (p<0.01) during the initial time (0 to 3 h) of incubation. However, NO_3_-N started disappearing rapidly at 3 h of incubation and its concentration was minimal in the batch culture and no difference was observed at 6 h of incubation. Differences in NO_3_-N concentration was observed at 12 and 24 h of incubation for nitrate or lactate supplementation. However, the difference does not seem to have biological meanings because the concentration of NO_3_-N was extremely low and the degree of the difference among treatments was trivial. The concentration of NO_2_-N was also low during the 24-h incubation for both nitrate and lactate supplementation, and accumulation of NO_2_-N was not observed in the current study. Although differences in NO_2_-N concentration was observed for lactate supplementation during the initial time of incubation (0 to 3 h; p≤0.02), the difference was extremely small. However, we observed that NO_3_-N concentration for Nit-Lact was numerically lower and was lower (p<0.01) at 3 h of incubation than that of Nit treatment. Along with this result, NO_2_-N concentration was greater (p<0.05) for Nit-Lact compared with that of Nit from 0.5 h to 3 h of incubation. Those results suggest that nitrate supplementation with lactate enhanced the rate of nitrate reduction compared with nitrate alone. Certain species of nitrate or nitrite-reducing bacteria are capable of utilizing lactate as a source of energy, for instance, *Selenomonas ruminantium* [15,29]. Iwamoto et al [29] also demonstrated that supplementation of electron donors, such as formate and lactate, stimulated nitrate and nitrite reduction in an *in vitro* batch culture. Therefore, the supplementation of lactate may have increased the activity or growth of nitrate-reducing bacteria, thereby accelerating the rate of nitrate reduction in the current study. However, although we observed the increased reduction of nitrate, it is not certain how much nitrate supplementation with lactate decreased the risk of nitrate toxicity compared with nitrate alone. Further *in vivo* studies are needed to determine their combination effect on nitrate reduction in the rumen and toxicity in an *in vivo* experiment. Nitrate and nitrite levels rose from 12 h of incubation for all treatments. This phenomenon may be attributed to accumulation of ammonia in the batch culture, and the nitrification process may have occurred due to high NH_3_-N concentration. de Melo Coelho et al. demonstrated that excessive ammonia concentration in the rumen may promote ruminal nitrification or denitrification, resulting in nitrous oxide (N_2_O) production observed in beef cattle [30].

## CONCLUSION

This study confirmed that nitrate supplementation was effective in decreasing methane production. However, nitrate supplementation also tended to decrease DM digestibility and decreased total gas production, showing its potential negative effects on feed fermentation in the rumen. However, the supply of nitrate with lactate eliminated the negative effect of nitrate alone on DM digestibility, suggesting potential benefits of feeding nitrate with lactate as a combination. Nitrate supplementation increased acetate production and decreased propionate, butyrate, and valerate productions, confirming that nitrate acted as an alternative electron sink in the rumen and resulted in methane mitigation. Furthermore, nitrate reduction to nitrite was enhanced when nitrate was provided with lactate compared with nitrate alone, which could be a potential health benefit when nitrate is used as a feed additive for the purpose of methane mitigation in cattle. It warrants further *in vivo* studies to confirm our results.

## Figures and Tables

**Table 1:** Ingredients and chemical composition of the basal diet

Items	Basal diet
Ingredients (% of dry matter)	
Corn silage	27.0
Alfalfa silage	29.6
Soyhulls	8.60
Corn grain (ground)	15.4
Soybean meals	15.2
Fat^1)^	1.80
Trace mineral premix^2)^	0.59
Minerals and vitamins^3)^	1.86
Chemical composition (% of dry matter)	
Dry matter (DM)	52.7
Organic matter (OM)	92.4
Crude protein (CP)	17.5
Neutral detergent fiber (NDF)	30.0
Starch	21.5
Total fatty acid	4.96
Ca	0.82
P	0.42

1) Spectrum Fusion, Perdue AgriBusiness Salisbury, MD, USA.

2) The premix contained (as-is basis) 39.3% sodium, 53.2% chloride, 70 mg/kg cobalt, 400 mg/kg copper, 70 mg/kg iodine, 1,750 mg/kg iron, 2,800 mg/kg manganese, and 3,500 mg/kg zinc (Morton Salt Inc., Chicago, IL, USA).

3) Mineral and vitamin contents were as follows (% of dietary DM): limestone = 0.91%; magnesium oxide = 0.07%; magnesium sulfate = 0.18%; calcium phosphate (di) = 0.48%; copper sulfate = 0.002%; zinc sulfate = 0.007%; selenium premix (200 mg/kg) = 0.13%; vitamin A = 0.01%; vitamin D = 0.036%; vitamin E = 0.046%.

**Table 2:** Effects of nitrate and lactate supplementation on dry matter digestibility, total gas and methane production during *in vitro* incubation

Item	Hour	Treatments^1)^				SEM	p-value^2)^		
	
		CON	Nit	Lact	Nit-Lact		Nitrate	Lactate	Int
Dry matter (DM) digestibility (%)	24	45.3^ab^	42.2^b^	44.3^ab^	46.6^a^	4.89	0.78	0.21	0.06
Total gas production (mL)	3	95.3	94.4	98.8	91.2	4.82	0.21	0.96	0.32
	6	191	176	183	176	16.8	<0.01	0.09	0.11
	12	269	259	263	246	17.1	<0.01	<0.01	0.13
	24	314	295	311	297	17.6	<0.01	0.67	0.14
Methane production (mL)	3	4.90	4.40	5.50	4.30	0.29	<0.01	0.34	0.13
	6	14.9	11.1	14.2	12.0	1.93	<0.01	0.86	0.10
	12	24.7	22.1	24.8	20.2	2.30	<0.01	0.23	0.21
	24	31.0	25.3	31.0	25.8	1.71	<0.01	0.46	0.43

1) CON, control; Nit, nitrate; Lact, lactate; Nit-Lact, nitrate and lactate.

2) Nitrate, main effect of nitrate; Lactate, main effect of lactate; Int, interaction of nitrate by lactate.

a,b Within a row, means without a common superscript letter differ (p<0.05).

SEM, standard error of the mean.

**Table 3:** Effects of nitrate and lactate supplementation on nitrate and nitrite nitrogen during *in vitro* incubation

Item	Hour	Treatments^1)^				SEM	p-value^2)^		
	
		CON	Nit	Lact	Nit-Lact		Nitrate	Lactate	Int
NO_3_-N (mg/L)	0	0.00^b^	76.3^a^	0.00^b^	77.7^a^	2.81	<0.01	0.73	0.73
	0.5	0.00^b^	73.8^a^	0.00^b^	76.1^a^	5.41	<0.01	0.72	0.72
	1	0.00^b^	70.5^a^	0.00^b^	67.0^a^	5.34	<0.01	0.55	0.55
	3	0.00^c^	51.0^a^	0.00^c^	41.2^b^	5.42	<0.01	0.14	0.14
	6	0.05	0.07	0.07	0.07	0.052	0.46	0.43	0.31
	12	0.06^b^	0.07^b^	0.11^a^	0.07^b^	0.014	0.14	0.06	0.06
	24	0.12^a^	0.07^b^	0.12^a^	0.07^b^	0.030	0.01	0.94	0.75
NO_2_-N (mg/L)	0	0.018	0.017	0.020	0.021	0.001	0.96	0.02	0.51
	0.5	0.018^b^	0.018^b^	0.022^a^	0.024^a^	0.001	0.27	<0.01	0.33
	1	0.018^c^	0.022^b^	0.022^b^	0.025^a^	0.002	<0.01	<0.01	0.80
	3	0.023^b^	0.024^b^	0.030^ab^	0.036^a^	0.005	0.31	<0.01	0.37
	6	0.030	0.028	0.028	0.036	0.005	0.26	0.24	0.07
	12	0.156	0.152	0.152	0.149	0.051	0.56	0.62	0.99
	24	0.125	0.131	0.125	0.129	0.019	0.25	0.33	0.35

1) CON, control; Nit, nitrate; Lact, lactate; Nit-Lact, nitrate and lactate.

2) Nitrate, main effect of nitrate; Lactate, main effect of lactate; Int, interaction of nitrate by lactate.

a–c Within a row, means without a common superscript letter differ (p<0.05).

SEM, standard error of the mean.

**Table 4:** Effects of nitrate and lactate supplementation on ruminal pH, ammonia nitrogen and volatile fatty acids during *in vitro* incubation

Item	Hour	Treatments^1)^				SEM	p-value^2)^		
	
		CON	Nit	Lact	Nit-Lact		Nitrate	Lactate	Int
pH	3	6.59	6.58	6.58	6.59	0.011	0.91	0.91	0.43
	12	6.31	6.34	6.36	6.38	0.131	0.29	0.08	0.95
	24	6.23	6.28	6.26	6.26	0.104	0.24	0.77	0.29
NH_3_-N (mg/L)	3	69.4	44.4	69.6	46.5	14.43	<0.01	0.67	0.74
	12	190	169	180	173	23.1	0.08	0.63	0.36
	24	287	267	290	275	4.8	<0.01	0.13	0.55
Total VFA (mmol/L)	3	94.2	97.2	106.4	94.9	20.92	0.61	0.55	0.39
	12	137	150	135	145	15.75	0.11	0.63	0.84
	24	152	155	169	166	16.3	0.97	0.06	0.66
Acetate (%, mol/mol)	3	61.0	63.2	60.0	61.5	3.52	0.02	0.08	0.66
	12	56.6	60.7	56.4	59.3	1.57	<0.01	0.20	0.37
	24	54.8	59.4	55.0	57.0	0.82	<0.01	0.46	0.34
Propionate (%, mol/mol)	3	25.3	24.4	26.0	25.0	2.30	<0.01	0.04	0.92
	12	25.8	24.2	25.5	24.4	1.06	<0.01	0.97	0.30
	24	26.1	24.8	25.7	25.1	0.55	<0.01	0.77	0.17
Iso-butyrate (%, mol/mol)	3	0.41	0.42	0.38	0.43	0.066	0.06	0.67	0.30
	12	0.66	0.62	0.65	0.65	0.114	0.16	0.22	0.12
	24	0.77	0.75	0.76	0.76	0.125	0.70	0.77	0.64
Butyrate (%, mol/mol)	3	10.4	9.3	10.8	10.1	0.63	0.02	0.10	0.54
	12	13.1	11.2	13.4	11.9	0.40	<0.01	0.13	0.50
	24	13.8	12.0	14.0	12.8	0.74	<0.01	0.29	0.43
2-methyl butyrate (%, mol/mol)	3	0.34	0.27	0.28	0.29	0.034	0.45	0.48	0.29
	12	0.46	0.43	0.45	0.44	0.060	0.17	0.96	0.37
	24	0.61	0.59	0.60	0.60	0.062	0.66	0.94	0.56
Iso-valerate (%, mol/mol)	3	0.55	0.52	0.42	0.51	0.079	0.55	0.16	0.19
	12	0.73	0.67	0.71	0.69	0.109	0.10	0.98	0.32
	24	0.86	0.82	0.83	0.83	0.100	0.53	0.80	0.51
Valerate (%, mol/mol)	3	1.76	1.76	2.12	1.93	0.100	0.32	0.01	0.30
	12	3.52	3.33	3.86	3.72	0.270	0.07	<0.01	0.81
	24	4.59	4.03	5.29	4.75	0.153	<0.01	<0.01	0.92

1) CON, control; Nit, nitrate; Lact, lactate; Nit-Lact, nitrate and lactate.

2) Nitrate, main effect of nitrate; Lactate, main effect of lactate; Int, interaction of nitrate by lactate.

SEM, standard error of the mean; VFA, volatile fatty acid.
